# Radiation-Induced Transformation of Immunoregulatory Networks in the Tumor Stroma

**DOI:** 10.3389/fimmu.2018.01679

**Published:** 2018-07-26

**Authors:** Inigo Martinez-Zubiaurre, Anthony J. Chalmers, Turid Hellevik

**Affiliations:** ^1^Department of Clinical Medicine, Faculty of Health Sciences, UiT the Arctic University of Norway, Tromsø, Norway; ^2^Institute of Cancer Sciences, Beatson West of Scotland Cancer Centre, University of Glasgow, Glasgow, United Kingdom; ^3^Department of Radiation Oncology, University Hospital of Northern Norway, Tromsø, Norway

**Keywords:** radiotherapy, tumor microenvironment, immunotherapy, tumor stroma, angiogenesis, extracellular matrix, mesenchymal cells, myeloid cells

## Abstract

The implementation of novel cancer immunotherapies in the form of immune checkpoint blockers represents a major advancement in the treatment of cancer, and has renewed enthusiasm for identifying new ways to induce antitumor immune responses in patients. Despite the proven efficacy of neutralizing antibodies that target immune checkpoints in some refractory cancers, many patients do not experience therapeutic benefit, possibly owing to a lack of antitumor immune recognition, or to the presence of dominant immunosuppressive mechanisms in the tumor microenvironment (TME). Recent developments in this field have revealed that local radiotherapy (RT) can transform tumors into *in situ* vaccines, and may help to overcome some of the barriers to tumor-specific immune rejection. RT has the potential to ignite tumor immune recognition by generating immunogenic signals and releasing neoantigens, but the multiple immunosuppressive forces in the TME continue to represent important barriers to successful tumor rejection. In this article, we review the radiation-induced changes in the stromal compartments of tumors that could have an impact on tumor immune attack. Since different RT regimens are known to mediate strikingly different effects on the multifarious elements of the tumor stroma, special emphasis is given to different RT schedules, and the time after treatment at which the effects are measured. A better understanding of TME remodeling following specific RT regimens and the window of opportunity offered by RT will enable optimization of the design of novel treatment combinations.

## Introduction

Radiation therapy (RT), either used alone or combined with systemic therapies, is a cornerstone of cancer treatment. Technological improvements now enable precise delivery of large radiation doses to tumors, stimulating profound changes in RT treatment schedules for some cancers. The use of stereotactic body radiotherapy (SBRT), in which high-dose radiation is delivered with extreme precision in small numbers of fractions, is becoming increasingly widespread (1). RT impacts upon both tumor and host cells, exerting multiple effects beyond the simple destruction of malignant cells. In recent years, we have witnessed an increased awareness of the role played by the complex tumor microenvironment (TME) in the response to therapy (2, 3). Consequently, recent research has investigated the effects of radiation on tumor stroma elements such as fibroblasts, connective tissue, vasculature, or immune cells.

The field of cancer immunology has also witnessed tremendous progress, leading to the development of new therapies that do not target tumor cells but instead boost the host immune system to fight against malignancy. The clinical implementation of novel immunotherapies in the form of immune checkpoint inhibitors (ICIs) is becoming one of the greatest advancements in the history of cancer treatment (4). In responders, ICIs may induce long-lasting tumor regression, even in patients with multiple metastatic lesions (5). Recently, the immune contexture of the TME was introduced as a new concept that classifies tumors by quantifying immune cell densities, and may define the likelihood of responding to immunotherapy (6). Patients with lymphocyte-rich “hot” tumors have been seen to respond remarkably well to ICI with long-lasting tumor regression. Unfortunately, the majority of patients present with “cold” tumors, which may explain the relatively low response rates observed when ICI is given as monotherapy.

Radiotherapy has been proposed as a promising, readily available, non-toxic, and cost-effective partner to immunotherapy. The immune-stimulatory properties of RT have generated widespread interest based on preclinical and clinical observations that localized RT can induce regression of non-irradiated metastases (abscopal effects) (7). However, it remains to be determined whether radiotherapy is only an occasional enhancer of ICI effects or represents a true “game changer” (8). In addition, our understanding of how, and how often, radiotherapy can convert tumors from being unresponsive to responsive is limited. As a proof-of-principle, it was demonstrated more than 30 years ago that T-cells can contribute to radiation-induced tumor control, a phenomenon that adds to the direct killing of malignant cells (9, 10). Moreover, it has been shown that radiation is able to ignite adaptive antitumor immune responses through the induction of immunogenic cell death and the release of endogenous adjuvants from dying tumor cells (11, 12). Likewise, systemic antitumor responses after combined ICI and local RT have been demonstrated in some murine models (13–15). Nevertheless, abscopal effects of RT in the clinic remain rare, thus highlighting the need to better understand and address the obstacles to effective *in situ* tumor vaccination.

Numerous reports have demonstrated that the “*in situ* vaccination” effects of local radiotherapy are mediated through induction of immunogenic cancer cell death and the associated release of powerful danger signals, which are essential to recruit and activate dendritic cells (DCs) and mount an adaptive immune response. However, efficient immune rejection is often hindered by intrinsic barriers within the TME (16). For instance, migration of effectively primed T-cells into the tumor can be inhibited by the disorganized vasculature, high interstitial fluid pressure, and other mechano-biological and chemotactic signals. In addition, resident and recruited cells (and molecules) in the TME can impair the survival, activation, proliferation, and effector-function of cytotoxic T-cells. Given the importance of the multifactorial immunosuppressive forces encountered in the TME, in this review we focus on RT effects on stromal elements that may influence antitumor immune responses. Intentionally, we will not cover RT effects on the malignant component of tumors, which have been comprehensively reviewed by other authors in the past (17, 18).

In our view, insufficient consideration has been given to the divergent biological effects elicited either by different radiation regimens, or to the timing of key biological processes. Most preclinical studies exploring the immunogenic effects of RT (alone or in combination with immuno checkpoint blockers) have been limited to testing a single radiation dose or schedule at a single time point, despite the unquestionable fact that different radiation regimens induce markedly different cellular and tissue responses (2, 18). In addition, the numerous ongoing clinical trials exploring RT-IT combinations are not consistent with each other, and are largely designed based on empirical choices of radiation regimens instead of rational ones (19). Consequently, the outcomes are likely to be divergent and/or inconclusive, and may fail to demonstrate the ability of radiation to synergize with immunotherapy. In this review, therefore, we put special emphasis on describing effects associated with specific radiation regimens, and draw attention to the chronology of events. To avoid misinterpretation, we refer to radiation doses of 2 Gy or less as “low,” doses of 4–10 Gy as “intermediate,” and doses above 10 Gy as “high.”

## Effects of RT on ECM Remodeling, Conductivity, and Tissue Stiffness

Solid tumors generally display increased tissue stiffness and tensile strength compared to neighboring normal tissues. Tumor stiffening results from augmented deposition of interstitial extracellular matrix proteins, mainly collagen (fibers), but also hyaluronan, elastin, and fibronectin, along with a steadily increasing population of non-malignant and malignant cells. The mechanical forces mediated by these structural components (20) constitute physical barriers that hinder access and motility of blood-borne antitumor T-cells (21, 22), (therapeutic) antibodies (23), liposomes, and nanoparticle drugs (24), thereby greatly affecting immune surveillance and immunotherapy responses.

### Dynamic RT Effects on ECM Remodeling

Based on the idea that depletion or reduction of intratumoral collagen can reduce solid stress and open up compressed blood and lymphatic vessels (25), several laboratories have demonstrated improved blood-borne drug delivery by reducing collagen content (25–27). Paradoxically, RT, despite being a well-known trigger of fibrotic tissue reactions (28–31), has been shown to augment tumor penetration by “large” macromolecules such as monoclonal antibodies (32–34), and also liposomes, and nanoparticles (35–39), enhancing the passive processes of enhanced permeability and retention (40). The clue to understanding this paradox is time. Obviously, temporal aspects of drug/antibody administration versus RT delivery are of utmost importance in achieving optimal responses. The limited time-frame for using RT to improve drug distribution was highlighted by Jain et al. (29), who measured the effects of ionizing radiation (IR) (1 × 10 Gy) on tumor hydraulic conductivity, hyaluronan, and collagen type-I in colon adenocarcinoma xenograft tumors. They found unchanged collagen levels 24 h post-RT, but 4 days later hydraulic conductivity was decreased (12-fold) while collagen-I levels were elevated. Lower radiation doses may not induce such fibrotic reactions. In a preclinical study by Appelbe et al., quantification of collagen in xenograft tumors excised 17 days post-RT revealed increased collagen-I staining after high (1 × 15 Gy) but not low or moderate radiation doses (2 and 5 Gy) (38).

Enhanced intra- and inter-molecular cross-linking of collagen and elastin fibers is another factor directly affecting tissue stiffness. The enzyme lysyl oxidase (LOX), which initiates cross-linking in the extracellular space, is elevated in response to hypoxic microenvironments and various cytokines (41, 42), and is associated with metastasis and poor survival in breast and head-and-neck cancer (43). Inhibition of LOX activity decreased levels of fibrillar collagen, increased tumor infiltration of macrophages and neutrophils, eliminated metastases in models of orthotopic breast (43) and transgenic pancreatic cancer (44), and enhanced drug delivery in a PDAC tumor model (44). Of note, IR promotes secretion of LOX from several tumor cell lines in a time- and dose-dependent manner (45). Shen et al. analyzed conditioned medium from lung tumor cells collected 16–20 h after exposure to single RT doses (2, 5, or 10 Gy), and observed increased secretion of both active LOX enzyme and inactive LOX pro-enzyme, with 10 Gy increasing LOX secretion 15-fold. Histological quantification in irradiated lung tumor xenografts revealed no change after 24 h, but prominent changes in LOX were observed 48 h post-RT for the two regimens examined (1 × 10 Gy) and (2 × 10 Gy). Moreover, LOX blood serum levels 48 h post-RT were doubled in mice that received (2 × 10 Gy) compared to the group receiving (1 × 10 Gy) (45). Others have collected murine lung tissue 2, 4, 8, and 20 weeks after thoracic radiotherapy (5 × 6 Gy), and found elevated LOX expression and activity at every time point (46). *Time* post-RT is clearly an important factor to consider.

### The Role of Transforming Growth Factor Beta (TGF-β)

Radiation-induced fibrotic reactions are initiated and sustained by a cascade of pro-inflammatory cytokines, which are released hours to days after radiation exposure (28). TGF-β—a master switch for the fibrotic program (47)—stimulates collagen production and functions as a chemoattractant for fibroblasts, with the capacity to reprogram fibroblasts into tumor-promoting and *fibrosis-associated* myofibroblasts (48). Rube et al. irradiated the thoracic region of fibrosis-sensitive mice and examined temporal aspects of TGF-β expression. They found a dose-dependent induction of TGF-β in lung tissue: a single dose of 12 Gy triggered TGF-β release that peaked after 12 h, whereas 6 Gy released minor amounts of TGF-β (49). In a similar experiment, Finkelstein et al. found upregulated TGF-β during 14 days (50). In line with the notion that TGF-β is critical for radiation-induced fibrosis, blocking TGF-β reduces the fibrosis induced by high-dose RT in animal models (51, 52). In a mouse model of mammary carcinoma, Liu et al. blocked TGF-β and found decreased collagen content and normalized tumor interstitial matrix, which improved drug uptake and decreased tumor growth (25). Besides the well-known immune-suppressive functions exerted on inflammatory and immune cells, TGF-β modulates ECM deposition and tissue stiffness, thus exerting both direct and indirect immunoregulatory effects. TGF-β could therefore represent a major obstacle to radiotherapy-induced antitumor immunity, which may be overcome by TGF-β neutralizing antibodies (53). TGF-α may also be involved in radiation-induced lung injury, as elevated tissue levels of TGF-α (46) post-RT have been demonstrated.

### Dynamic Effects of RT on Proteases of the ECM

Connective tissue homeostasis is tightly controlled by the balanced expression of proteases and their inhibitors. Matrix metalloproteinases (MMPs) and their endogenous inhibitors, TIMPs, are key matrix regulators. Studies *in vitro* and *in vivo* have demonstrated radiation-induced alterations in protease activity, which may lead to increased tumor invasion (54, 55). In particular, transient and dose-dependent upregulation of extracellular MMP-2 and MMP-9 have been observed in irradiated cell lines derived from pancreatic cancer (54), glioma (56), lung cancer (57, 58), melanoma (59), fibrosarcoma (55), and hepatocarcinoma (60).

Transient upregulation of various MMPs in response to IR has been characterized in many experimental settings. Speake et al. analyzed conditioned medium from a fibrosarcoma cell line (55), and demonstrated pro-MMP-2 and pro-MMP-9 levels to peak at 24 and 48 h post-RT, respectively, whereas others found MMP-2 secreted by lung tumor cells to peak at 12 h (58) or 24 h (57) post-RT. Co-culture systems—exemplified by glial and endothelial cells (ECs)—are also responsive to RT, with MMP-2 and MMP-9 levels being markedly elevated 72 h after irradiation (61). Stromal cells also contribute to release of proteases into the TME. Human lung tumor fibroblasts respond to single-high radiation doses (18 Gy), by reducing secretion of MMP-1 when measured 5 days post-irradiation, whereas MMP3 levels are enhanced and MMP2 unchanged at the same time point (62).

In an animal model of Lewis lung carcinoma, serial measurement of urinary MMP-2 revealed increasing levels during tumor growth, but reduced levels 6 days post-RT (2 × 20 Gy) (63). At the clinical level, Susskind et al. measured plasma levels of MMP-9 and TIMP-1 in lung and breast cancer patients and observed very high levels before initiation of fractionated radiotherapy (66 Gy, 2.0 Gy/fx), a sharp decline in MMP-9 levels within 10 days of completion of RT, but no change in TIMP-1 levels (64). The latter finding is in line with results from irradiated human lung tumor fibroblasts (62). IR also affects membrane-associated metalloproteinases (or ADAMs). McRobb et al. found that a single dose of 20 Gy to brain microvascular ECs downregulated the alpha secretase ADAM10, with concomitant upregulation of ADAM10 target proteins at the cell surface (65). Another study by Sharma et al. revealed that radiotherapy activates ADAM17 in non-small cell lung cancer (NSCLC), inducing shedding of multiple survival factors, growth factor pathway activation, and IR-induced treatment resistance (66).

**Figure 1:**
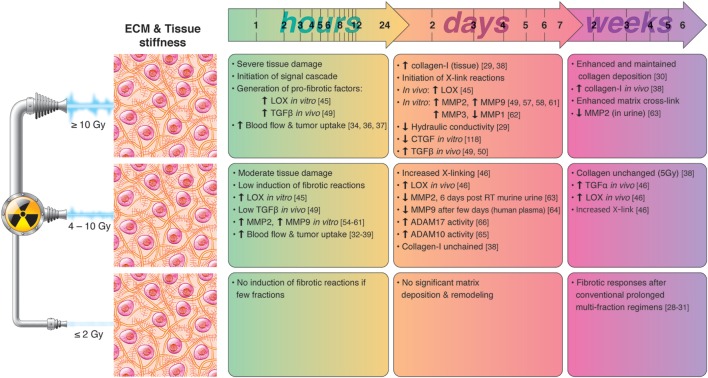
Chronological effects observed on ECM remodeling agents and tissue stiffness after RT delivered at different dose per fraction. *The figure is a compilation of observations registered in different experimental models, comprising primary cell cultures (in vitro), cell lines, animal (mainly mice) models, and clinical observations, on effects of RT given at different dose/fraction. Radiation schemes range from single fractions to oligo (daily) fractions and prolonged multifractionated regimens. The vast majority of preclinical observations comprise RT regimens of one or few fractions irrespective of the radiation dose. In clinical settings, RT protocols comprising moderate or high doses are always applied in one or few fractions*. Although some inconsistencies may exist between studies, it is generally observed that small doses in few fractions do not ignite substantial changes in ECM composition and tissue stiffness, whereas both medium and high RT doses exert measurable changes on matrix deposition and tissue stiffness in a dose- and time-dependent manner. RT mediated pro-fibrotic effects and matrix stiffness may become apparent several days post-irradiation and may last for weeks and months after RT.

Collectively, these studies underscore the importance of tissue stiffness on drug uptake and immune cell infiltration. Lessons learned from the field of drug delivery indicate that RT can be used to transiently reduce intratumoral interstitial pressure and increase vascular permeability. However, the effects of RT are temporary and only provide a window of opportunity during the first day(s) after the radiation insult. By contrast, prolonged exposure to multiple fractions of RT seems to induce matrix deposition, long-term fibrotic reactions, and increased stiffness. A summary of radiation-induced effects on ECM remodeling and tissue stiffness is presented in Figure 1.

## Effects of RT on Tumor Vasculature and Lymphatic Vessels

Trafficking of newly activated antigen-specific T-cells is dysfunctional in cancers. Tortuous and leaky vessels hinder transit and extravasation of leukocytes into tumors; an imbalance of pro- and anti-angiogenic factors in solid tumors contributes to such vascular aberrations. The tumor vasculature is also a recognized obstacle to therapeutic access, and both preclinical and clinical studies have shown that vascular normalization can augment drug delivery in tumors. Such approaches may also enhance antitumor immunity.

### Dynamic RT Effects on Tumor Vessels

Effects of RT on blood endothelial cells (BECs) are highly dependent on total dose and fraction-size, as well as tumor stage-location-type and maturation stage of vessels. High-dose RT (≥10 Gy) is more likely to induce EC death (67) and tumor vessel collapse (68, 69), whereas at low doses (≤2 Gy), BEC survival is promoted through miRNA upregulation (70) with enhanced EC migration and angiogenesis (71). There is some evidence that intermediate doses (4–10 Gy) may induce tumor vessel normalization and vessel dilation, reducing vascular leakage and increasing tumor oxygenation (72, 73). Scheduling must also be taken into consideration if combination strategies are to be optimized. Kabacik and Raj found that endothelial permeability to macromolecules of various sizes increased in a radiation dose-dependent manner, and involved ADAM10 activation and cleavage of VE-cadherin junctions (74). Park et al. measured vascular permeability in the skin of C3H-mice exposed to local irradiation (2, 15, or 50 Gy), and found that it peaked 24 h post-IR, followed by a gradual decrease to baseline over the next 3–10 days. Of note, the extent and duration of vascular permeabilization was dose-dependent (75). Kalofonos et al. also measured vascular permeability and vascular volume of irradiated (1 × 4 Gy) colon adenocarcinoma xenografts (34), and observed increased vascular permeability 24 h post-RT, but no differences between treated and control tumors at 72 h. Appelbe et al. (38) applied an intermediate radiation dose (5 Gy) to mammary adenocarcinoma xenografts, with drug administration before and after RT, and demonstrated 1.2- to 3.3-fold enhancement of probe accumulation in tumors. In addition, they observed maintained vascular integrity during the first 2 days post-RT, even at doses up to 15 Gy. They concluded that intermediate to high doses of radiation—insufficient to achieve tumor control—are sufficient to enhance drug delivery, independent of endothelial integrity. Other authors have also observed that low to intermediate RT doses (≤5 Gy) can stimulate angiogenesis (71) and/or vasculogenesis (76) in ECs. Hallahan et al. measured microvascular blood flow in irradiated murine hind-limb tumors just before and 24 h after RT, and found that a single-low dose of 2 or 3 Gy increased tumor blood flow 24 h post-RT, whereas 6 Gy markedly reduced blood flow (77). Others have observed that a single dose of 8 Gy causes minimal damage to microvessels and the EC lining (78), with a modest 4.3% reduction in perfusion (4 h post-RT). Kolesnick et al. have previously suggested a threshold dose of (1 × 10 Gy) for induction of apoptosis in ECs (79).

While inconsistencies in the preclinical literature persist, accumulating evidences indicate that the main response of quiescent BECs to IR is induction of premature senescence rather than apoptosis (80). Panganiban et al. found that 10 Gy induced accelerated senescence in the majority of pulmonary artery ECs (87%, 120 h post-IR), but only residual levels of apoptosis (81). Moreover, at doses above 8 Gy, 99% of the ECs were alive but not competent to form colonies. Oh et al. irradiated bovine aortic ECs (5, 10, and 15 Gy) and observed increasing numbers of large, flattened senescent-like cells at higher doses, with a twofold increase in average cell surface area after 15 versus 10 Gy (67). Massive cell death appeared 2–5 weeks after 15 Gy, whereas 5 Gy induced only transient morphological disturbances. Others have also demonstrated radiation-induced senescence in BECs (82–84), with long-lasting DNA damage responses and durable nuclear foci formation (82, 84). Of note, the extent and duration of senescence in various types of BECs after different radiation doses corresponds with radiation-induced senescence in lymphatic endothelial cells (LECs) (85) and cancer-associated fibroblasts (CAFs) (62).

In general, extensive endothelial damage after doses above 10 Gy causes reduced vascular flow, which impairs effector T-cells recruitment to the tumor, and exacerbates the hypoxia-driven immunosuppressive environment. Hypofractionated regimens using doses per fraction below 10 Gy might induce sufficient cancer cell death without exacerbating hypoxia and immunosuppression.

### RT Effects on Cell Adhesion Molecules in ECs

Dysfunctional extravasation of leukocytes into tumors because of structural abnormalities of vessels is exacerbated by changes in the adhesive properties of tumor ECs. Reduced expression of E-selectin may lead to impaired lymphocyte recruitment. Other adhesion receptors such as ICAM-1, ICAM-2, and VCAM, which facilitate integrin-mediated extravasation, are often poorly expressed by tumor-associated ECs.

Radiation exposure is known to alter the expression of cell adhesion molecules on ECs. Hallahan and colleagues irradiated human umbilical endothelial cells (HUVECs) and observed induced expression of both E-selectin and ICAM-1 in a dose- and time-dependent manner (86). Threshold doses of 1 and 5 Gy for induction of E-selectin and ICAM-1, respectively, were observed, however, VCAM-1 and P-selectin surface expression were apparently unaffected by IR. Similarly, Gaugler et al. (87) irradiated cultured HUVECs and observed upregulation of ICAM-1 but not VCAM-1 after various doses of IR (2, 5, and 10 Gy). Others exposed epidermal keratinocytes and dermal microvascular ECs to 6 Gy, and found that IR triggered surface expression of ICAM-1 on these cells within 24 h, independent of *de novo* protein synthesis (88). At sub-lethal doses, IR may enhance expression of certain cell adhesion molecules in ECs and thereby contribute to leukocyte homing and immune recognition.

### Recruitment of Endothelial Progenitors Following RT

Vasculogenesis, the formation of new blood vessels by recruitment of bone marrow-derived endothelial precursor cells (BMDCs), is a major mechanism for vessel repair and tumor regrowth after RT (89). Several laboratories have demonstrated radiation-induced recruitment of proangiogenic myeloid BMDCs into tumors, orchestrated by chemotactic SDF-1-CXCR4 signaling. In an intracranial xenograft model of glioblastoma (GBM), Kioi et al. found that whole brain irradiation (8 or 15 Gy) triggered dose-dependent recruitment of BMDCs into tumors (90). Interestingly, BMDC levels were only slightly elevated from control levels after 8 Gy, but more than doubled after 15 Gy. However, BMDC influx and/or retention after 15 Gy was efficiently blocked by AMD3100, an inhibitor of the SDF-1/CXCR4 axis. In this study, AMD3100 was administered on the day of irradiation, with continued infusion over the following 21 days. Kozin et al. exploited the same concept in breast and lung tumor xenografts, and found that combined AMD3100 and local irradiation significantly delayed tumor growth, but only when the drug was applied immediately after local irradiation (91). In their model, drug administration 5 days post-IR was ineffective. Hence, radiation-induced recruitment of BMDCs into tumors was suggested to be a rapid process (91). Altogether, results from preclinical studies indicate that a single large dose of local irradiation may trigger two waves of BMDCs influx (92): one shortly after exposure (3–5 days) (91) and a second delayed response (associated with hypoxia) after about 2 weeks (90). Accumulated knowledge coming mainly from preclinical models supports the notion that recruitment of bone marrow precursors is the main mechanism behind tumor neovascularization following RT, and that the effect is proportional to the radiation dose. Importantly, this process seems to be activated immediately after radiation exposure and completed within few days after tissue damage.

### RT Effects on Pericytes

Pericyte coverage is also abnormal in tumor vessels; pericytes appear to be loosely associated with vessels and with poorly developed basal lamina, therefore contributing to increased leakiness. Increased VEGFA in the TME may hinder pericyte function and survival by suppressing PDGFRβ signaling. Pericytes from tissues such as the liver may also exert direct immunomodulatory effects by expressing negative co-stimulatory molecules (93) or, as in malignant glioma, by secretion of paracrine immunosuppressive signals, including PGE2, TGFβ, and NO (94).

The effects of radiation on pericytes have scarcely been investigated. In a xenograft model of neuroblastoma, tumor blood volume measurements 6 h post-RT were reduced by 63 and 24% after 12 and 2 Gy, respectively. Histopathological examination revealed a significant loss of EC at 6 and 12 h, and an additional loss of both mature and immature pericytes at 72 h (95). However, high-dose RT is postulated to enhance recruitment of mesenchymal stem cells to the TME, which could promote pericyte recovery and tumor recurrence. In a xenograft study by Wang et al. (96), bone marrow mesenchymal precursors were observed to home into tumors and transform into pericytes following (1 × 14 Gy) irradiation in an SDF-1 and PDGF-B-depending manner. Fractionated irradiation of murine prostate TRAMP-C1 tumors at intermediate doses (15 × 4 Gy) resulted in reduced microvascular density but increased tumor perfusion, associated with dilated vessels tightly connected to BM-derived pericytes (97). In a similar manner, Lewis lung carcinoma-bearing mice treated with high-dose RT (1 × 12 Gy) or (3 × 12 Gy) exhibited reduced microvessel density but increased perfusion, reduced hypoxia, and increased pericyte coverage (98).

Collectively, these studies suggest that irradiating tumors with both intermediate and high doses results in decreased microvascular density but increased perfusion due to dilation of surviving vessels and increased pericyte coverage, taking place some days after RT.

### RT Effects on Lymphangiogenesis

Lymphatic vessels constitute a transport route for both antitumor immune cells and metastatic spread of tumor cells. However, the disorganized lymphatic system that is characteristic of solid tumors can lead to impaired fluid flow and increased interstitial pressure (99). LECs may also hinder antitumor immunity by cross-presentation of tumor antigens in a VEGF-C-dependent manner (100). In addition, the lymphatic drainage of tumor antigens may affect antitumor immunity by promoting a tolerogenic environment in sentinel lymph nodes (100).

Despite the fact that lymph nodes and vessels are often included in the irradiated field in clinical practice, relatively few studies have explored the effects of IR on LEC integrity and function. An array of studies have documented that, contrary to blood vessels, high doses of RT (>10 Gy) do not affect lymphatic vessel integrity (101–103). In skin biopsies from breast cancer patients, similar numbers of lymphatic vessels were observed in irradiated and non-irradiated sites (103). Sung et al. examined responses to high-dose radiation on LECs in the small intestine of adult and embryonic mice and in peri-tumoral areas of mice, and concluded that intestinal and peri-tumoral LECs are highly resistant to radiation-induced apoptosis (102). In fact, LECs are likely to respond to IR by the induction of stress-induced cellular senescence. Avraham et al. exposed cultures of dermal LECs to single doses of 4, 8, or 12 Gy and found that (4 days post-IR) senescence was triggered in 53, 64, and 74% of the cell population, respectively (85). The same study revealed a minor 8% apoptosis-induction in LECs upon (1 × 15 Gy). A recent study by Rodriguez-Ruiz et al., which utilized cultures of primary human LECs as well as mouse transplanted tumors and pre- and post-RT patient samples (104), revealed a radiation-dose and time-dependent induction of ICAM-1 and VCAM-1 surface expression on LYVE-1+ LECs. The maximum effect was observed at 20 Gy and persisted for more than 8 days. The authors proposed that such an effect may mediate enhanced adherence of T-lymphocytes on irradiated LECs.

**Figure 2:**
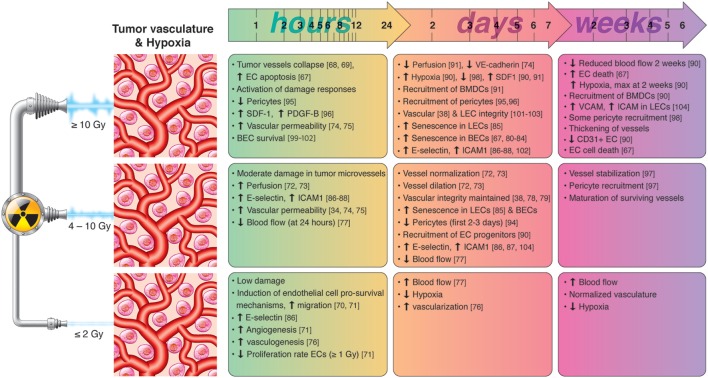
Chronological effects observed on tumor vasculature and hypoxia after RT delivered at different dose per fraction. *The figure is a compilation of observations registered in different experimental models, comprising cell cultures (in vitro), animal (mainly mice) models, and clinical observations, on effects of RT given at different dose/fraction. Radiation schemes range from single fractions to oligo (daily) fractions and prolonged multifractionated regimens. The vast majority of preclinical observations comprise RT regimens of one or few fractions irrespective of the radiation dose. In clinical settings, RT protocols comprising moderate or high doses are always applied in one or few fractions*. Although some inconsistencies may exist between studies, it is generally observed that small doses in few fractions promotes endothelial cell (EC) survival and increased intratumoral blood flow, whereas high-dose RT induces EC apoptosis, hypoxia-response elements and the recruitment of EC progenitors during the first days post-RT. Medium radiation doses, given in one or few fractions, induce moderate damage in tumor blood vessels, promotes the dilation and normalization of existing vessels, pericyte recruitment, and the expression of cell adhesion molecules. Lymphatic endothelial cells (LECs) are more radioresistant than blood vessels but may enter into premature senescence already after moderate radiation doses.

Few reports studying normal tissue reactions to radiotherapy propose that IR at high doses may induce impairment of the lymphatic vasculature (105). However, most studies highlight the radioresistant nature of LECs and the beneficial effects of RT on induction of adhesion molecules that favor T-cell recruitment and extravasation. A summary of radiation-induced effects on tumor vasculature and hypoxias is presented in Figure 2.

## Mesenchymal Cells, Radiation, and Immunity

### RT Effects on CAFs

Immunomodulation is one of the best-characterized tumor regulatory mechanisms exerted by CAF. In general, CAFs are considered to promote an immunosuppressive TME. However, new evidence suggests that such effects may be specific for certain CAF subsets, and may depend on temporal and contextual factors (106, 107). Through secretion of a plethora of cytokines, chemokines, proteases, and proangiogenic factors, CAFs may exert both direct and indirect effects on tumor immunity. Direct effects on effector memory T-cells are mediated *via* secretion of potent immunoregulators such as TGFβ, PGE2, TSLP, interleukin (IL)-6, IL-8, or nitric oxide (16). In addition, CAFs may mediate indirect effects by expression of ECM molecules that attenuate antitumor immunity, such as tenascin-C, galectin-3, or thrombospondin-1, by participating in ECM synthesis and turnover, or by exerting an impact on tumor angiogenesis (108). Moreover, CAFs express cytokines and chemokines that support the recruitment and maintenance of immunosuppressive myeloid cells, promote the polarization of macrophages toward the M2-phenotype, and interfere with maturation of DCs (109). In the context of RT, CAFs are considered to be very radioresistant (62, 110–112), however, exposure to IR is able to induce cellular senescence in fibroblasts, especially at doses above 12 Gy (62). In xenograft models, senescent fibroblasts co-transplanted with cancer cells have been found to increase tumorigenicity. A recent preclinical study by Li and colleagues (113) demonstrated radiation (1 × 4 Gy) to enhance the tumor-promoting effects of CAFs, an effect that was associated with increased expression of CXCL12. However, the overall tumor regulatory properties of senescent or irradiated fibroblasts remain controversial, as other studies have observed no impact of (high-dose) irradiation on the tumor enhancing effects of fibroblasts, or even loss of pro-malignant properties (114–116).

The immunoregulatory phenotype of irradiated fibroblasts is less well characterized, since most *in vivo* studies have been conducted on immunocompromised animals. A recent *in vitro* study revealed that primary lung CAFs maintain their immunosuppressive phenotype after exposure to both high (1 × 18 Gy) and low (4 × 2 Gy) radiation doses (117). On the other hand, high dose IR (1 × 18 Gy) has been shown to alter the secretory profile of CAFs and the expression of factors that could exert immunomodulatory effects, directly or indirectly (118). Multiplex protein analyses on conditioned medium collected from irradiated human lung CAFs from five different donors with NSCLC revealed that single-high dose RT (1 × 18 Gy) leads to a prominent (38%) and significant reduction of SDF-1 and threefold reduction in macrophage inhibitory factor (118). Besides their direct paracrine effects on inflammatory and immune cells, CAFs may influence tumor immune responses indirectly by mediating ECM remodeling. As indicated earlier, CAFs are major contributors of desmoplastic reactions in tumors and thus could exert indirect effects on tumor immune infiltration by regulating tissue stiffness and interstitial fluid pressure. One recent study has compared levels of αSMA expressing CAFs in tumor specimens from colorectal cancer patients receiving neoadjuvant radio(chemo)therapy (45 Gy in 25 fractions) before and after treatment (119). Results from this study revealed increased amounts of αSMA expressing myofibroblasts and connective tissue post-therapy. Connective tissue growth factor (CTGF) is also mitogenic and chemotactic for fibroblasts, and stimulates synthesis of collagen-1 (33) and fibronectin (34). In response to IR (1 × 18 Gy), secreted levels of CTGF from human lung CAFs are reduced 3.5-fold compared to controls, suggesting that exposure to ablative radiation doses may exert anti-fibrotic effects on CAFs (118). However, in an animal model, *ex vivo* irradiated CAFs (1 × 18 Gy) co-implanted with A549 tumor cells induced tumors with similar extents of collagen deposition and inflammatory cell infiltration as tumors established with non-irradiated CAFs (116).

**Figure 3:**
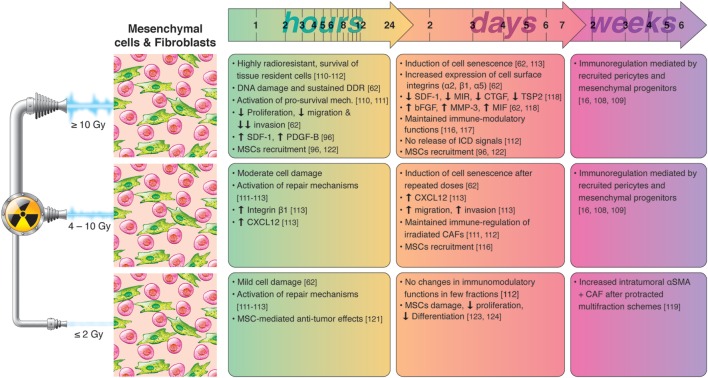
Chronological effects observed on cancer-associated fibroblasts (CAFs) and mesenchymal cells after RT delivered at different dose per fraction. *The figure is a compilation of observations registered in different experimental models, comprising primary cell cultures (in vitro), cell lines, animal (mainly mice) models, and clinical observations, on effects of RT given at different dose/fraction*. Studies on RT effects on CAFs are scarce. It is well demonstrated that tumor fibroblasts are quite radioresistant and may survive even ablative doses of radiation. Small doses may not change importantly the phenotype and functions of CAFs. At high doses, CAFs become senescent within few days post-irradiation, less motile, and less proliferative. High RT doses may substantially alter the secretory profile of CAFs characterized by reduced levels of SDF-1, MIR, or connective tissue growth factor (CTGF), and increased levels of bFGF, MMP3, or macrophage inhibitory factor (MIF), observed within few days post-RT. Of relevance, *in vitro* observations reveal that the immunosuppressive functions of cultured CAFs are preserved after exposure to high and low RT doses. In a long run, mesenchymal progenitors and pericytes recruited after medium and high doses may contribute importantly to immunoregulations, to prevent the onset of autoimmune reactions.

Recognizing that we still lack knowledge on the effects mediated by irradiated CAFs in the tumor context, and that different CAF subtypes may respond differently to IR, overall the existing literature indicates that CAFs are likely to survive radiation insults and that high-dose irradiation could exert beneficial effects in relation to CAF-mediated tumor immune regulation. A summary of radiation-induced effects on fibroblasts and immuno-regulation is presented in Figure 3.

### Effects of RT on Mesenchymal Stromal Cells (MSCs)

Tissue damage provoked by RT triggers the recruitment of MSCs from distant reservoirs such as bone marrow or adipose tissue. Recruited MSCs post-treatment have been associated with both pro- and antitumorigenic effects. The migration and differentiation potential of MSCs were characterized in a Lewis lung carcinoma and malignant melanoma-bearing recipient mice treated with (SB)RT, 14 Gy/1 fraction (96). Recruitment of circulating MSCs was promoted by secretion of SDF-1 and PDGF-B from irradiated tumor cells. In this study, it was proposed that engaged MSCs transform into pericytes to promote tumor vasculogenesis and tumor regrowth. On the contrary, irradiated MSCs may be a source of antitumor cytokines that decrease the proliferative activity and induce apoptosis of tumor cells (120). In the study by de Araújo Farias et al. (121), *in vivo* administration of unirradiated mesenchymal cells together with radiation lead to an increased efficacy of radiotherapy. In a separate study, tumor irradiation was shown to enhance the tumor tropism of adoptively transferred human umbilical cord blood-derived mesenchymal stem cells in an IL-8-dependent manner (122). Enhanced therapeutic effects were associated to TRAIL delivered by MSCs.

The effects of RT delivered in low-dose multifraction schedules on MSCs can be more unpredictable. MSCs recruitment may start already after the first cycles of radiation, however, IR, even when delivered at low doses, can have profound effects on the biology of MSCs. In a recent *in vitro* study, bone marrow-derived MSCs isolated from normal adults were irradiated with 2 Gy twice daily for consecutive 3 days (123). Irradiated MSCs showed much lower proliferative and differentiation potential, and induced clonal cytogenetic abnormalities of MSCs. Likewise, when isolated MSCs were irradiated with 2 Gy alpha particles or X-rays, adverse effects were observed on the vitality, functionality, and stemness of MSCs (124).

Collectively, efforts in this field have shown that RT, especially when delivered at high doses, triggers the recruitment of progenitor mesenchymal cells into the irradiated tumors, and that such recruitment could exert both tumor-promoting or tumor-inhibiting effects. Considering the demonstrated immunoregulatory potential of MSCs, recruited MSCs following RT could play an important role on immunomodulation, however, this particular hypothesis remains to be demonstrated experimentally.

## Effect of Local Radiation on Inflammatory Cells

Myeloid-derived cells are an important part of the TME, both numerically and functionally, and play central roles in regulating tumor vasculature and antitumor immune responses. Myeloid cells arise from a common myeloid progenitor that, upon differentiation, gives rise to various cell types including tumor-associated macrophages (TAMs), DCs, polymorphonuclear neutrophils, and myeloid-derived suppressor cells (MDSCs). Myeloid cells in tumors may exist in various differentiation stages, and possess a susceptible immunomodulatory phenotype that can be influenced by radiation.

Radiation-mediated changes on myeloid cells include killing of tumor-associated pools, recruitment of circulating progenitors, repolarization, and reorganization (125). Of note, bone marrow-derived cell recruitment following RT involves mainly SDF-1/CXCR4–7, CCL2/CCR2–4, and colony-stimulating factor-1 (CSF-1)/CSF-1R pathways. Observed effects seem to depend on radiation regimens and the timing post-RT, however, pre-existing tumor microenvironmental parameters such as hypoxia, necrosis, pH, stroma composition, and cytokine milieu may all influence tumor leukocyte composition following RT.

### RT Effects on Macrophages

Tumor-associated macrophages are considered to be relatively radioresistant because of their well-developed anti-oxidative machinery. However, IR is able to affect both phenotype and recruitment of TAMs. Globally, data generated in different tumor types and using different RT regimens indicate that high doses (10–30 Gy)—either as single dose or oligo-fractioned (≤3×)—trigger recruitment of CD11b+ myeloid cells and reprogramming of macrophages toward the tumor-promoting M2-phenotype (126, 127). Interestingly, selective ablation of CD11b+ or CD18+ cells (128), or blockage of the SDF-1/CXCR4 or CSF-1/CSF-1R pathways prevents accumulation of myeloid cells/macrophages and improves antitumor immune response and the overall response to IR (90, 129). Of importance, upregulation of the M2-gene signature has been observed within few days of irradiation and may last for several weeks or even longer (130, 131). In the TRAMP-C1 prostate cancer model, a single fraction of 25 Gy or 15 fractions of 4 Gy induced the M2-genes COX2 and Arg-1 within few days (126). On the contrary, intermediate radiation doses (2–5 Gy) given in few fractions have been reported to repolarize macrophages from M2- to the pro-immunogenic M1-phenotype *in vitro* and *in vivo*. Non-polarized, monocyte-derived macrophages established in cultures shifted toward the M1-phenotype after daily (5 × 2 Gy) radiation schemes (132). Doses of 5–10 Gy have been shown to increase nitric oxide synthase and decrease M2-phenotypic traits (133). *In vivo* experiments have mainly utilized small doses. Klug and colleagues demonstrated that single fractions of (0.5–2.0) Gy polarize macrophages toward the iNOS + M1-phenotype (134), whereas whole body irradiation with a single dose of 2 Gy caused CD11+ peritoneal macrophages to repolarize into the M1-phenotype. In another study, induction of the M1-phenotype in tumors after local IR (1 × 2 Gy) was only possible in combination with CD8+ T-cell transfer (134). Upon M1 repolarization, the resulting iNOS expression appears to be responsible for vascular normalization, T-cell recruitment and activation, and finally tumor rejection. Of note, very low radiation doses (under 1 Gy) may favor the M2-phenotype of TAMs, as evidenced by *in vitro* culture experiments performed with different macrophage sources (135–137).

In summary, the accumulated knowledge in this area postulates that high-dose irradiation or moderate doses in multiple fractions facilitate the recruitment and reprogramming of macrophages with immunosuppressive functions, and that medium and low-dose radiation (down to 1 Gy) in single or few fractions may elicit immune-stimulatory macrophages that could help to unlock barriers to immunotherapy responses.

### RT Effects on MDSCs

As with macrophages, local radiation is able to mobilize other myelomonocytic CD11b+ cells with immunosuppressive functions in tumors. MDSCs have the unique ability to radioprotect tumor cells through expression of high levels of Arginase-I, with subsequent depletion of l-arginine from the microenvironment, a common mechanism behind T-cell and macrophage inhibition (138). Many and varying effects of radiation on mobilization and function of MSDCs have been reported and are likely to be influenced by the pre-existing systemic and local immune contexture. As described for macrophages, several studies in murine models have reported increased recruitment of MDSCs after high-dose RT. In a glioma model, high-dose radiation (1 × 15 Gy) induced more marked recruitment of CD11b+ myeloid cells than lower doses (1 × 8 Gy) (90). In addition, selective inhibition of CSF-1/CSFR-1 signaling was observed to improve the efficacy of RT by reducing recruitment of immunosuppressive MDSCs (129). Low radiation doses may exert different effects. Whereas human subjects treated with protracted RT regimens show elevated CSF-1 in peripheral blood, analyses of immune cell composition in peripheral blood of patients receiving fractionated chemoradiotherapy often reveal a reduction in both MDSCs and Tregs in relation to effector T-cells after treatment (139–142). A study comparing intratumoral infiltration of immunocytes pre- and post-neoadjuvant chemoradiotherapy in rectal cancer specimens demonstrated significant elevation of CD8+ and CD4+ T-cells post-treatment whereas MDSC, Tregs, and expression of co-inhibitory receptors remained stable (143). Similarly, ablative radiotherapy (1 × 30 Gy) has been shown to increase CD8+ cells and decrease MDSC in the TME of CT26 and MC38 murine tumors, whereas fractionated radiation did not trigger such strong lymphocytic responses (144).

### RT Effects on DCs

Dendritic cells can be divided into several subsets with specialized functions, and are key intermediaries between the innate and the adaptive immune systems. However, very few studies have documented the effects of RT on DC subsets and their roles in immune regulation.

Previous work have shown that DCs are relatively resistant to IR and exhibited limited changes in response to high-dose irradiation, such as upregulation of CD80 and reduced levels of IL-12 but not IL-10 (145). The effect of IR on phagocytosis and antigen presentation in DCs appears to depend on radiation dose and DC maturation state. For instance, 5 Gy gamma irradiation downregulated expression of co-stimulatory receptors CD80/CD86 on immature derived DCs but not on mature DCs (146). In a different study, CD86 expression was increased in immature but decreased in mature DCs after 30 Gy, while other markers remained unaffected (145). Of interest, in the former study, irradiation impaired the stimulatory effects of both mature and immature DCs on proliferation of allogeneic T-cells (145). Although *in vitro* studies suggest that IR compromises the stimulatory activities of DCs, *in vivo* models demonstrate that IR at intermediate radiation doses (5 × 8.5 Gy) enhances the ability of DCs to capture tumor antigens, and promotes DCs migration to lymph nodes in a toll-like receptor-dependent manner (147, 148). A number of studies have demonstrated increased presentation of tumor antigens by DCs in the tumor-draining lymph nodes after RT. For example, in B16-OVA and B16-SIY melanoma models, single radiation doses (15–25 Gy) or five fractions of 3 Gy increased the number of antigen-presenting cells cross-presenting tumor-specific antigens, which correlated with increased priming of antitumor T-cell responses (149, 150). It is important to note that *in vivo* effects mediated by recruited “non-irradiated” DCs may explain the discrepancies between *in vitro* and *in vivo* observations.

Of importance, IR effects on DCs can also differ between murine and human systems. At a dose of 0.2 Gy, ϒ-irradiation increased surface expression of CD80, CD86, MHC-class I and II receptors in murine DCs, but inhibited their capacity for antigen uptake. In addition, this low-dose IR suppressed IL-12 production and increased IL-10, implying a shift to immune tolerance (151). On the other hand, low-dose radiation under 1 Gy did not affect surface markers or cytokine production in either immature or mature human DCs, and had no influence on the capacity of DCs to stimulate T-cell proliferation (152).

Different radiation schedules may influence DC function and recruitment in different ways. In a murine melanoma study testing intratumoral DC vaccination, it was demonstrated that (5 × 8.5 Gy) enhanced the ability of DCs to capture tumor antigens without inducing enhanced DC maturation, but improving cross-priming of T-cells (147). Hypofractionated RT has been shown to recruit and activate DCs, however, this effect maybe time-restricted. In a recent preclinical study using colon cancer as a model, MHC-II positive DC recruitment into tumors was observed only between days 5 and 10 after the first radiation dose (153). In patients, conventional low-dose multifraction regimens may have detrimental effects on DCs. In head-and-neck cancer patients, neoadjuvant treatment was associated with a general decrease of tumor infiltrating DCs in intraepithelial compartments as assessed by IHC (154). In a study from Liu et al., authors found a significant decrease of BDCA3+ DCs, the immune-stimulatory variant, in the blood of patients treated with conventional radiotherapy (155).

The majority of *in vitro* studies indicate that moderate and high radiation doses are able to inhibit antigen presentation capacity and production of Th1 cytokines by DCs. However, *in vivo* studies seem to reflect opposite effects. DCs responses to RT can be very divergent between hypofractionated (SBRT) or multifraction regimens. To understand the contradictory observations published in this area, it is utterly important to consider the difference between tumor-associated DC pools that become irradiated during treatment (normally occurring during long-lasting conventional RT) versus non-irradiated DCs that infiltrate tumors after treatment (possibly occurring in SBRT strategies).

**Figure 4:**
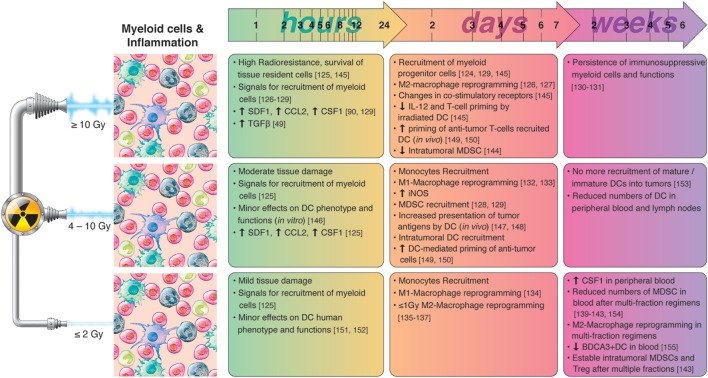
Chronological effects observed on myeloid cells and inflammation after RT delivered at different dose per fraction. *The figure is a compilation of observations registered in different experimental models, comprising primary cell cultures (in vitro), cell lines, animal (mainly mice) models, and clinical observations, on effects of RT given at different dose/fraction. Radiation schemes range from single fractions to oligo (daily) fractions and prolonged multifractionated regimens. The vast majority of preclinical observations, using cell cultures and animals, comprise RT regimens of one or few fractions irrespective of the radiation dose. In clinical settings, RT protocols comprising moderate or high doses are always applied in one or few fractionations*. In small doses, RT do not affect substantially the phenotype and function of dendritic cells (DCs). However, such doses have been shown to promote M1-polarization of macrophages and monocyte recruitment. The severe tissue damage provoked by high radiation doses induces the rapid release of chemotactic molecules such as CCL2, colony-stimulating factor-1 (CSF1) and SDF-1, and the recruitment of myeloid cells. High radiation doses induce M2 polarization of macrophages, impair the immune-stimulatory functions of tissue resident DCs, and activates de recruitment of myeloid-derived suppressor cell (MDSC). Medium radiation doses also trigger the recruitment of myeloid cells to tumors, however, immune-activating effects preponderate, characterized by M1-polarization of macrophages, and increased presentation of tumor antigens by DC. The mobilization of myeloid cells following RT cease after the first week(s), and it is normal to observe reduced numbers of MDSC, monocytes, or DCs in peripheral blood when prolonged multifraction regimens are completed.

A summary of radiation-induced effects on myeloid cells and inflammation is presented in Figure 4.

## Concluding Remarks

A considerable number of ongoing clinical trials are aiming at improving the efficacy of immune checkpoint blockers by local radiotherapy. Mounting evidences reveal that RT may prime and/or induce tumor-specific adaptive immune responses through the induction of immunogenic cell death the release of tumor-specific antigens and danger signals, and the ignition of an inflammatory cascade. However, it is still uncertain whether RT can be used effectively to enhance the effects of immunotherapeutic drugs in clinical settings. In fact, radiation may promote immunosuppressive reactions in several ways, such as upregulation of co-regulatory molecules PD-L1 and PD-L2 (156, 157), transient potentiation of hypoxia, or by recruiting and reprogramming of immunosuppressive myeloid cells. Treatment outcomes will ultimately depend on the net effect of pro-immunogenic and anti-immunogenic signals, and will be heavily dependent on pre-existing host and tumor factors. Moreover, even after defining optimal RT regimens for combinatory treatments, numerous physical and functional barriers to immune attack must be overcome to achieve clinical benefit. These include immunosuppressive elements in the stromal components of non-irradiated metastasis, and antigenic heterogeneity at different metastatic sites.

The effects of radiation on the multifactorial elements of the TME may be tumor type and tumor stage specific, may be influenced by the pre-existing tissue contexture, and are likely to be highly dependent on the treatment protocol. In this review, we have attempted to gather existing knowledge on the potential effects exerted by different radiation schemes in the compartments of the tumor stroma that may modulate antitumor immunity. Published studies range from *in vitro* experiments to preclinical *in vivo* models and clinical observations. Despite intense endeavors, most of the existing preclinical reports are limited to exploring effects of a single radiation dose or regimen. The treatment outcomes reported could be equally influenced by experimental variables such as the intrinsic immunogenicity and/or radiosensitivity of the tumor cells, the immune competence of the host, implantation site, and tumor stage. Thus, information gathered from preclinical studies should not be interpreted as universal dogmas or generalizable evidences with direct applicability in the clinics. Also, knowledge from clinical studies is limited because of the inherent restrictions associated with the clinical protocols, where, for example, immunological effects are normally measured from peripheral blood samples and only rarely in the irradiated tissues. Conclusion about the relative effects of different radiation schemes on immune activation can only be made by performing systematic comparisons using the same tumor model.

Although the existing knowledge is fragmented, model-specific and in some cases inconsistent, some key patterns emerge. In general, high-dose RT, given as single dose or in few fractions, results in severe tissue damage, increased tumor cell death, and enhanced release of tumor-associated antigens and related danger signals. However, high-dose RT also seems to activate mechanisms that counterbalance these potentially overwhelming immune reactions. Thus, downstream effects associated with high-dose RT comprise substantial damage to tumor vasculature, transient potentiation of hypoxia, increased fibrosis and interstitial pressure, recruitment and reprogramming of immunosuppressive myeloid cells, and release of signals that favor Th2 pathways. On the contrary, low-dose radiation protocols (2 Gy/fraction and below) are often followed by a number of immune adjuvant effects comprising normalization of tumor vasculature, enhanced expression of cell adhesion molecules, increased perfusion, decreased interstitial fluid pressure and reprogramming of tumor infiltrating macrophages into the antitumorigenic M1-phenotype. However, low-dose RT may not be very effective in boosting the generation of tumor-associated antigens and danger signals. Furthermore, the conventional clinical protocols based on multifraction regimens applied over several weeks may exert detrimental effects on recruited DCs and effector T-cells, thus hampering the establishment of tumor-specific immune responses. Intermediate radiation dose protocols seem to reproduce many of the positive effects observed with low radiation dose protocols, including vessel normalization and transient induction of pro-inflammatory environments. Hypofractionated regimens comprising doses per fraction below 10 Gy might generate meaningful levels of cancer cell death without exacerbating hypoxia and immunosuppression. However, to achieve responses that can synergize with immunotherapies, it is of the utmost importance to consider time and treatment sequence. In many instances, immune adjuvant effects occur within hours of RT treatment, and may be maintained for only a few days before the favorable circumstances are changed or lost. In such circumstances, radiation should perhaps be applied in reduced number of fractions, concomitant with or immediately after administration of the immunotherapeutic drug has begun.

For the future, we encourage clinicians and scientists to use existing knowledge to design clinical trials for assessing the overall clinical benefit of radiation combinations, and employ rational choices of dose, fractionation, treatment sequence, and timing. In parallel, further mechanistic studies are needed to understand how dose and fractionation influence the effects of RT on the pre-existing TME. There is a need to systematize protocols and knowledge by designing comparative studies of different RT-schemes using unmodified and immune competent animal models. The use of radiotherapy as a partner for immunotherapy is an exciting and revolutionary concept, but much remain to be learned before its true clinical potential is realized.

## Author Contributions

IMZ and TH contributed equally to the initial conception, the development and the writing of the article. AJC has critically revised the manuscript for important intellectual content and has approved the final version to be published.

## Conflict of Interest Statement

The authors declare that the research was conducted in the absence of any commercial or financial relationships that could be construed as a potential conflict of interest.
